# Enhancing cucumber production through compost and plant growth promoting rhizobacteria in an unheated soil based greenhouse

**DOI:** 10.1038/s41598-026-36907-2

**Published:** 2026-01-30

**Authors:** İbrahim Memelİ, Yüksel Tüzel, Tunç Durdu, Mahmut Tepecİk, Nazim S. Gruda

**Affiliations:** 1 Department of Horticulture, Faculty of Agriculture, Ege University, Bornova, 35100 İzmir, Türkiye; 2 Department of Soil Science and Plant Nutrition, Faculty of Agriculture, Ege University, Bornova, 35100 İzmir, Türkiye; 3 Department of Horticultural Sciences, University of Bonn, INRES-Institute of Crop Science and Resource Conservation, 53113 Bonn, Germany

**Keywords:** *Bacillus subtilis*, Compost dose, *Pseudomonas fluorescens*, Greenhouse cucumber, Principal component analysis (PCA), Ecology, Ecology, Environmental sciences, Microbiology, Plant sciences

## Abstract

The transition toward more sustainable horticultural practices requires approaches that maintain productivity while reducing environmental impact. The use of organic amendments such as compost and plant growth-promoting rhizobacteria (PGPR) offers environmentally friendly strategies to enhance soil health, nutrient availability, and plant resilience. Here, we investigated the effects of different compost doses and PGPR inoculation on plant growth, yield, and fruit quality of greenhouse-grown cucumber, cv. ‘Oscar’. Compost was applied to the upper 10 cm depth of soil at four rates (0 g m^−2^, 100 g m^−2^, 200 g m^−2^, 300 g m^−2^) and two PGPR strains, *Bacillus subtilis* and *Pseudomonas fluorescens*, were applied twice to the root zone. Correlation analysis and Principal Component Analysis were used to evaluate associations between growth parameters, yield, and leaf nutrient status. The interaction between compost and PGPR significantly increased root fresh and dry weight. The most effective treatment—200 g m^−2^ compost combined with *Pseudomonas fluorescens*—enhanced marketable yield by 9.3% relative to the untreated control. Yield improvements were closely linked to increased nutrient uptake, particularly magnesium (Mg) and phosphorus (P), as confirmed by PCA. The highest nutrient enrichment was observed with the 300 g m^−2^ compost + *Pseudomonas fluorescens* treatment, where Mg and P increased by 13.5% and 27%, respectively, compared to the control. Overall, the combination of compost at 200 g m^−2^ with *Pseudomonas fluorescens* represents a practical and sustainable strategy to improve cucumber performance under greenhouse conditions. Future research should assess its effectiveness across diverse soil types, stress conditions, and production systems to optimize long-term implementation.

**Supplementary Information:**

The online version contains supplementary material available at 10.1038/s41598-026-36907-2.

## Introduction

 Increasing urban populations impose significant pressure on agricultural productivity and the environment, resulting in unsustainable land-use practices that accelerate soil degradation and pollutant mobilization^1^. Moreover, chemical pollutants originating from agricultural activities are projected to infiltrate freshwater ecosystems and designated conservation areas, negatively impacting water resources by reducing both their availability and quality^2,3^.

The recognition that intensive chemical input practices irreversibly compromise soil, water, and food safety has heightened the importance of sustainable production methods^4,5^. Soil health and fertility management are fundamental components of sustainable agriculture, underpinning crop productivity, environmental sustainability, and the long-term resilience of agricultural production systems^6^.

Soil organic matter delivers multiple benefits: it prevents nutrient leaching while stabilizing soil pH; improves physical and chemical properties through enhanced cation exchange and water-holding capacity; maintains soil structure through aggregation; provides nutrients to crops and soil organisms; and neutralizes soil-damaging pollutants^7,8^. Increasing soil organic matter content significantly enhances soil fertility, thereby restoring ecological balance. Organic fertilizers, particularly compost, serve as the primary source for this improvement while enabling the utilization of organic waste from various activities^9–11^. Contemporary understanding of agricultural sustainability has elevated the importance of organic/biological fertilizers, with compost gaining particular prominence.

Organic fertilizers provide superior benefits compared to chemical fertilizers across plant, soil, environmental, and economic dimensions^12–15^, while promoting soil microbial activities^16,17^. In cucumber cultivation specifically, compost encourages plant growth, limits the uptake of organic contaminants^18^, and serves as a sustainable component of growing media^19^.

Beneficial microorganisms residing in plant root zones, including Arbuscular Mycorrhizal Fungi (AMF)^20,21^, Plant Growth-Promoting Rhizobacteria (PGPR)^22,23^, and symbiotic Rhizobia^24,25^. They represent safe practices for plant, soil, and environmental health in agricultural production^26–28^. The importance and utilization of microbial preparations have increased substantially in recent years, with microbial fertilizers becoming essential agricultural inputs for enhancing production, protecting soil fertility, and increasing crop yields^29–33^. The value market size, projections, and annual growth rates reflect growing commercial interest in these technologies^34,35^.

Previous cucumber cultivation studies have examined various organic fertilizer applications, including green manuring^36^, vermicompost^37,38^, and chicken manure combined with mineral fertilizers^39^. Research has demonstrated that beneficial soil microorganisms, such as *Bacillus* and *Pseudomonas* species, enhance disease suppression^40^. Additionally, the co-application of organic and mineral fertilizers can produce favorable results^41,42^.

However, studies on the combined application of beneficial microorganisms with compost remain limited. Research has primarily focused on individual components, with studies examining compost rates of 75–125% of the recommended nitrogen dose, either with or without the addition of a PGPR mixture or compost tea^43^. At the same time, synergistic studies report promising results with microbial composts developed by incorporating characterized plant-growth-promoting bacteria or fungal strains into compost, or by suppressing diseases^44,45^. There are still critical knowledge gaps regarding soil- and crop-specific standardized compost application rates, the strain-specific effectiveness of *Bacillus subtilis* versus *Pseudomonas fluorescens* across varying soil conditions, and the relationships between plant growth and plant nutrient content.

With this study, we aimed to evaluate compost doses and PGPR as sustainable alternatives in a protected drinking-water basin where strict restrictions on synthetic inputs require compliant, environmentally safe production systems. We hypothesized that compost doses and PGPR treatments, namely *Bacillus subtilis* or *Pseudomonas fluorescens*, would enhance plant growth and yield while enabling us to discern the specific effects of nutrient contributions.

The objectives were to improve yield through the use of different compost doses and *B. subtilis* or *P. fluorescens*, increase leaf nutrient uptake—particularly nitrogen and phosphorus—, demonstrate the relationships among root and shoot biomass, leaf nutrient contents, and yield parameters in grafted cucumber production, and to clarify whether there is a synergistic effect of compost and the tested PGPR.

## Materials and methods

This study was conducted in the summer of 2021 in a polyethylene greenhouse at an altitude of 160 m above sea level in the vicinity of Tahtalı Dam Protection Basin in the Çamönü neighborhood of Menderes district, Izmir province (38° 11’ 30.11“ N, 27° 16’ 62.45” E). The research greenhouse was a traditional unheated multispan greenhouse having an arc-shaped roof, an iron main frame, polyethylene cover, and a multispan roof with a width of 24 m, a length of 72 m, a side height of 2.2 m, and a roof height of 4.0 m, located in the east-west direction.

### Planting material, compost, and PGPR

‘Oscar F1’ (Multi Seed Company, Antalya/Türkiye) cucumber cultivar, which is suitable for tunnel and greenhouse cultivation during spring and summer seasons, having medium-strong, green, slightly ribbed skin fruits and high tolerance to powdery and downy mildew^46^, was preferred as plant material. Vitalley F1 (Syngenta), having a high growth rate and disease resistance (Co: 1/Fon: 1, 2/Fom: 0, 1, 1–2, 2), was used as rootstock^47^. Grafted seedlings were obtained from Ege Fide Company (Izmir, Türkiye).

Ecorec Environment and Energy Technologies Company Limited produced Ecorec compost organic fertilizer (Izmir, Türkiye), which was used in this research. Ecorec compost organic fertilizer was created by converting waste products into organic compost fertilizer through various processing steps. The physicochemical properties of the organic compost fertilizer and greenhouse soil used in the experiment are given in Table 1.

**Table 1:** Physicochemical properties of Ecorec organic compost fertilizer (Anonymous, 2025) and greenhouse soil.

Parameter	Compost	Soil
pH	7.3	7.73
Salinity (EC) dS m^−1^	2.07	1.6
Composition	–	Clayey loam
Organic matter (%)	35.7	3.41
Total nitrogen (%)	3.6	0.084
Total phosphorus (mg kg^−1^)	16,000	8.12
Total potassium oxide (K_2_O) (mg kg^−1^)	18,000	403.8
Calcium (Ca) (mg kg^−1^)	89,000	4756.8
Magnesium (Mg) (mg kg^−1^)	10,000	164.6
Iron (Fe) (mg kg^−1^)	10,000	5.21
Manganese (Mn) (mg kg^−1^)	200	6.86
C/N ratio	12.36	
Total (humic + fulvic) acide	28.6%	

Greenhouse soil was analyzed in the laboratory of Ege University, Faculty of Agriculture, Department of Soil Science and Plant Nutrition.

*Bacillus subtilis* (Serenade SC), one of the beneficial root bacteria used in the study, was obtained from Bayer Turkish Chemical Company Limited (Istanbul, Türkiye). The active substance was 1.34% *B. subtilis* QST713 strain (1 × 10^9^ cfu ml^− 1 48^). The other beneficial root bacteria used in the study, *Pseudomonas fluorescens* strain Pf1 (1.5%, 1 × 10^8^ cfu ml^− 1^ min.) (Cedriks SL), was obtained from Agrobest Group Agriculture and Trade Company (İzmir, Türkiye).

### Treatments

A total of 384 plants were planted in 12 treatment plots (4 compost doses × 3 PGPR treatments), with 4 replicates (8 plants per replicate) for each treatment, each measuring 100 × 50 × 50 cm. The area of each plot was calculated as 10 m². A spacing of 1 m between plots was maintained^49^. Compost was applied to the soil before planting by manually spreading the specified dose over the plots and subsequently mixing it into the top 10 cm of soil using a hoe. Compost material “Ecorec” organic compost (CMP) was used in 4 doses (CMP0: 0 g m^− 2^, CMP100: 100 g m^− 2^, CMP200: 200 g m^− 2^, CMP300: 300 g m^− 2^) based on the company’s recommendations, as well as our previous results in lettuce^50^ and mixed into the soil. *B. subtilis* (Serenade SC) (Bac) and *P. fluorescens* (Cedriks SL) (Pse) were applied to the root zone during planting and 2 weeks later. In the root zone of the seedlings planted in the plots where compost was applied, 8 ml Pse (6.5 × 10^5^ cfu ml^− 1^) and 11 ml Bac (6.5 × 10^6^ cfu ml^− 1^) per plant were applied with a syringe from the beneficial root bacteria solution prepared as 65 ml Pse per 1 L of water and 65 ml Bac per 1.5 L of water. The process was repeated after 2 weeks as 1 × 10^7^ cfu/ml Bac and 3 × 10^5^ cfu ml^− 1^ Pse per plant. The untreated plots (RB0: no rhizobacteria; CMP0) served as controls. The research was conducted using a 4 × 3 factorial randomized block design (*n* = 4). For statistical analyses, mean values calculated for each plot were considered as the experimental units.

### Cultivation technology used

After the land was tilled, solarization was applied. Based on the soil analysis results, fertilization followed^51^ guidelines aligned with a yield target of 300 t ha^−1^. All phosphorus, along with 22% of nitrogen and 33% of potassium, was applied as basal fertilizer before planting. The remaining nitrogen and potassium were supplied via daily fertigation throughout the growing period^52^. The production period began with seedling planting and continued from July to November. Planting was carried out at a rate of 2.66 plants per square meter. Since the cucumber plants tend to wind, they were tied up, and shoot pruning was carried out. Cultural practices were employed for disease and pest management in the greenhouse, and when necessary, recommended licensed pesticides were used to control pests. Harvesting was done when the fruit reached 16–18 cm in length, which is a characteristic of the variety.

### Measurements and analysis

At the end of the harvest season, before uprooting, three plants were randomly selected from each plot for each treatment, and their roots were carefully removed, washed, and cleaned. Then the fresh weight of the roots was determined by weighing them with a precision balance, and the dry weight was determined after drying in an oven at 65 °C for 48 h. The fresh and dry weights of the upper parts were determined by repeating the process. Final values were calculated by dividing the results by the number of samples^53^.

The total yield was calculated as the yield per plant by weighing the fruits collected from the replicate plants on each harvest day, from the first to the last, and then dividing by the number of plants in the replicate. The number of fruits was recorded with each harvest, and the total number of fruits per plant was determined at the end of production. Average fruit weight was calculated by dividing the total yield by the total number of fruits.

In October, the color of five randomly selected fruit samples from each replicate was measured using a Minolta CR-300 colorimeter in L*a*b* mode from three different points to represent the entire color. In the chromometer, the L* value indicates brightness, the a* value indicates color changes from red to green, and the b* value indicates color changes from yellow to blue^54^.

At the end of the eighth week, pH values of fruit juice were measured with a pH meter (Mettler Toledo - SevenGo, Giessen, Germany), and total soluble solids (TSS) were determined using an Abbe digital hand refractometer (Euromex RD 645, Arnhem, The Netherlands) on the filtered juice obtained from 3 randomly selected fruits that were shredded in a blender^55^. 10 mL of cucumber juice from the same fruit was titrated with 0.1 N sodium hydroxide (NaOH) using a pH meter until the pH reached 8.01. The titratable acid (TA) was calculated from the amount of NaOH consumed^56^. Five randomly selected fruits were peeled from different points of the fruit (middle and near the two ends), and the firmness was measured in kg as a result of pressure with a penetrometer from these peeled parts^57^.

On October 21, 2021, samples were taken from the 4th- to 5th-fully expanded young leaves, starting from the growth tip downward. Sampling was conducted only once during the experiment to monitor nutrient content under the various treatments^58^. The samples were cleaned with distilled water, dried in an oven at 65 °C for 48 h, and ground in a mill to prepare them for analysis.

For plant nutrient analysis, total nitrogen (N) was determined using the Kjeldahl method, as described by Cepuliene et al. ^59^. Other nutrients were determined spectrophotometrically using the vanadomolybdate phosphoric yellow color method after wet digestion (HNO3:HClO4, 4:1). K and Ca were measured by flame photometry, and Mg, Fe, Zn, Mn, and Cu were analyzed by an Atomic Absorption Spectrophotometer^53^.

### Data evaluation

The effects of the treatments on plant growth, yield, fruit quality, and nutrient content were visualized using a heatmap generated with the online clustvis package^60^. Before clustering, the data were standardized using unit-variance scaling (z-score transformation), with each variable centered at zero and scaled to a standard deviation of 1. For hierarchical clustering, the correlation distance-based similarity measure and the average linkage method were applied to^61^. The data were graded using an artificial color scale, showing that red values increased and blue values decreased. Analysis of variance was applied to the study data using JMP 8^62^, and differences between averages were determined using the Tukey test at the 5% significance level. We indicated that the ANOVA assumptions were confirmed before analysis. Specifically, the data met the assumptions of normality, verified using the Shapiro-Wilk test, and homogeneity of variances, verified using the Levene test. PCA analysis was performed using JMP Pro 17^63^. Using the scores and loadings obtained from PCA, a biplot was created using the R program, with genotypes represented as points and variable loadings as arrows. The correlation matrix visualisation was performed using the R programme^64^ with the ggplot2^65^ and reshape2^66^ packages.

## Results

### Fresh and dry weights of root and shoot

**Fig. 1:**
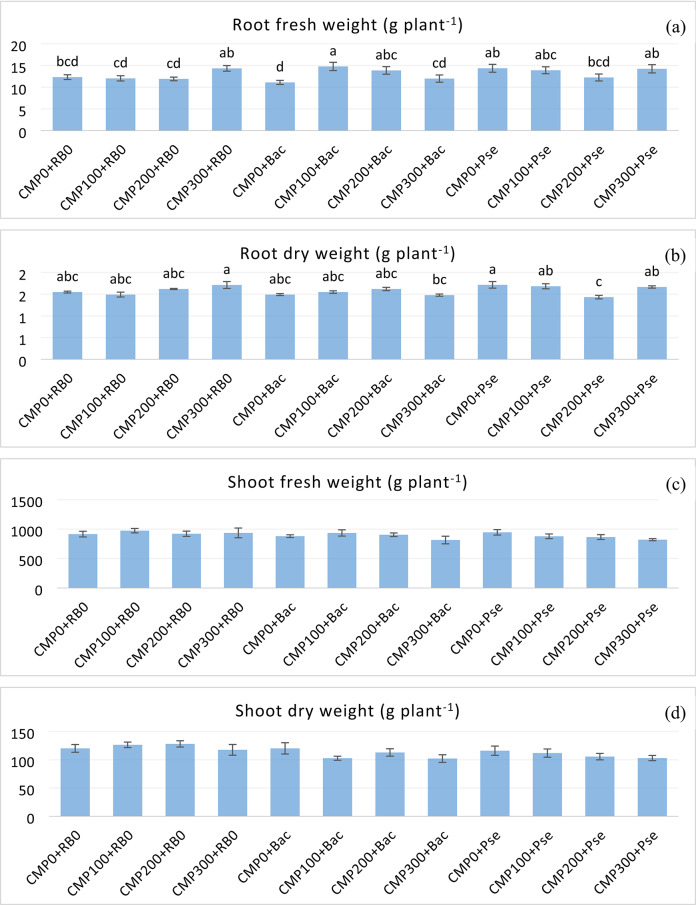
Effects of compost doses and PGPR treatments on root fresh weight** (**a**), root dry weight** (**b**), shoot fresh weight^ns^ (**c**), shoot dry weight^ns^ (**d**) of grafted cucumber. (Statistical significance: *P < 0.05; **P < 0.01; ns = not significant; letters show significant differences in mean values of treatments). Bars represent mean ± SD (*n* = 4). CMP0: 0 g m^− 2^ compost; CMP100: 100 g m^− 2^ compost; CMP200: 200 g m^− 2^ compost; CMP300: 300 g m^− 2^ compost; RB0: No PGPR; Bac: *Bacillus subtilis*; Pse: *Pseudomonas fluorescens*.

The treatments impacted root growth. The interaction between compost doses and plant growth-promoting rhizobacteria on root fresh and dry weights was statistically significant. Root fresh weight was highest in the CMP100 + Bac (14.79 g plant^−1^), representing a 33% increase compared with the lowest treatment, Bac without compost (Fig. 1a). The average root dry weight was higher in the Pse treatments. Root dry weight was highest in the CMP0 + Pse and CMP300 + RB0. Increasing compost doses had a positive impact on root dry weight, even in the absence of rhizobacteria (Fig. 1b).

The effects of the treatments on the fresh and dry weight of the shoots were not significant, and the dry matter content of the shoots was generally higher in the compost treatment (including the control). The fresh weight of shoots varied between 815 g plant^− 1^ and 974.08 g plant^− 1^ (Fig. 1c), and the dry weight of shoots varied between 102.08 g and 128 g plant^− 1^ (Fig. 1d).

### Leaf element contents

**Fig. 2:**
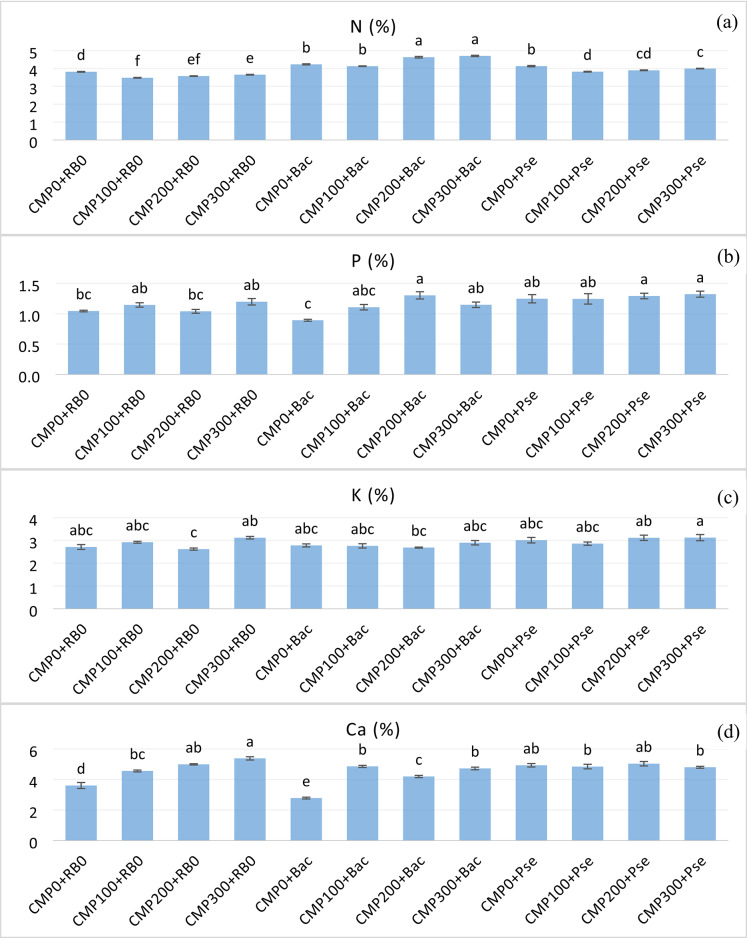

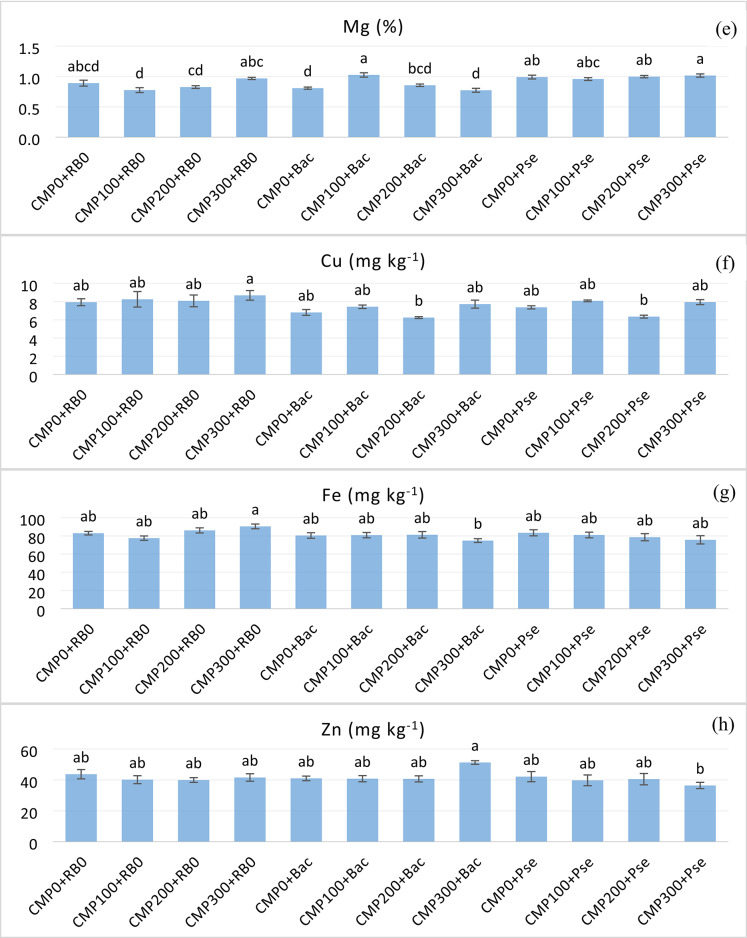
Effects of compost doses and PGPR treatments on N** (**a**), P** (**b**), K** (**c**); Ca** (**d**); Mg** (**e**); Cu** (**f**), Fe* (**g**), and Zn* (**h**) content of leaves of grafted cucumber. (Statistical significance: *P < 0.05; **P < 0.01; ns = not significant; letters show significant differences in mean values of treatments). Bars represent mean ± SD (*n* = 4). CMP0: 0 g m^−2^ compost; CMP100: 100 g m^−2^ compost; CMP200: 200 g m^−2^ compost; CMP300: 300 g m^−2^ compost; RB0: No PGPR; Bac: *Bacillus subtilis*; Pse: *Pseudomonas fluorescens*.

The main and interaction effects of compost doses and root bacteria on the N, P, K, Ca, and Mg contents of leaves, excluding the main impact of compost doses on Mg, were found to be statistically significant, as determined from leaf samples collected from the experimental treatments. The highest N content was recorded in the CMP300 + Bac (4.70%) and CMP200 + Bac (4.63%) treatments, indicating a positive effect of higher compost doses combined with Bac (Fig. 2a). The average N content of Bac was 21.8% and 11.6% higher than RB0 and Pse, respectively. CMP300 exhibited the highest N content.

P, K, and Mg contents were higher in Pse and at higher compost doses. P and K contents were the highest in CMP300 + Pse (Fig. 2c). Leaf mean Ca content was higher in CMP100 and CMP200. Mean Ca value was 5 and 18% higher in Pse compared with Bac and RB0 (Fig. 2d). Mg content of leaves was found to be high in *P. fluorescens*. The highest values in the interaction were CMP100 + Bac (1.03%) and CMP300 + Pse (1.02%), respectively (Fig. 2e).

The main and interaction effects of compost doses and root bacteria on Cu, as well as the interaction effects on the Fe and Zn contents of leaf samples, were significant. Cu content of leaves varied between 8.69 (CMP300 + RB0) and 6.24 (CMP200 + Bac) ppm (Fig. 2f). Cu and Fe patterns sometimes peaked in non-PGPR treatments. Fe content was the highest in the CMP300 + RB0 treatment. The lowest Cu and Zn were detected in CMP300 + Bac (Fig. 2g) and in CMP300 + Bac (Fig. 2h), respectively.

### Yield and fruit quality

Harvests began in early September and continued until mid-November. A total of 23 harvests were conducted over the 8-week yield period, with 2 or 3 per week. The main and interaction effects of compost doses and root bacteria on total yield, total fruit number, and average fruit weight were found statistically significant.

While the total yield was the highest in Pse, CMP200 yielded the highest among the compost doses. When the interaction effect was analysed, the highest yield was observed with CMP200 + Pse (9.41 kg plant^−1^), CMP300 + Pse, and CMP200 + Bac, while the lowest was observed with CMP100 + RB0 (6.73 kg plant^−1^) (Table 2). CMP200 + Pse increased the yield by 9.3% compared with CMP0 + RB0 (without compost and rhizobacteria).

**Table 2:** Effects of treatments on total yield (kg plant^−1^).

	RB0	Bac	Pse	MEAN(CMP)
CMP0	8.61 abc	7.14 cd	8.75 ab	8.17 B
CMP100	6.73 d	8.82 ab	8.73 ab	8.09 B
CMP200	8.31 abc	8.99 a	9.41 a	8.90 A
CMP300	8.38 abc	7.46 bcd	9.10 a	8.31 AB
MEAN(RB)	8.01 B	8.10 B	8.99 A	

Lower-case letters indicate the difference between means in interaction effects, and upper-case letters indicate the difference between means in main effects. (P values: for total yield; MEANcmp- 0.0156, MEANrb- 0.0002, Int- 0.0002). CMP0: 0 g m^− 2^ compost; CMP100: 100 g m^− 2^ compost; CMP200: 200 g m^− 2^ compost; CMP300: 300 g m^− 2^ compost; RB0: No PGPR; Bac: *Bacillus subtilis*; Pse: *Pseudomonas fluorescens*.

As in total yield, total fruit number, and average fruit weight were found to be the highest in the Pse treatment. When the interaction effect was analyzed, the highest total fruit number was observed in CMP200 + Pse, CMP100 + Bac, CMP300 + Pse, CMP0 + Pse, CMP100 + Pse, and CMP200 + Bac (Table 3). When the impact of interactions on average fruit weight was analyzed, the highest values were observed in CMP200 + Pse (Table 4). Compared with the Bac and RB0 treatments, Pse increased total fruit number by 9.4% and 14.3%, and average fruit weight by 2.1% and 9%, respectively.

**Table 3:** Effects of treatments on the total number of fruits (pcs plant^− 1^).

	RB0	Bac	Pse	MEAN(CMP)
CMP0	63.04 ab	52.05 bc	65.89 a	60.33 A
CMP100	49.37 c	67.65 a	64.09 a	60.37 A
CMP200	59.5 abc	63.79 a	67.80 a	63.62 A
CMP300	59.87 abc	58.73 abc	67.20 a	61.93 A
MEAN(RB)	57.95 B	60.55 B	66.25 A	

Lowercase letters indicate differences between means in interaction effects, whereas uppercase letters indicate differences between means in main effects. (P values: for total fruit number; MEANcmp- 0.2546, MEANrb- < 0.0001, Int- < 0.0001). CMP0: 0 g m^− 2^ compost; CMP100: 100 g m^− 2^ compost; CMP200: 200 g m^− 2^ compost; CMP300: 300 g m^− 2^ compost; RB0: No PGPR; Bac: *Bacillus subtilis*; Pse: *Pseudomonas fluorescens*.

**Table 4:** Effects of treatments on average fruit weight (kg plant^− 1^).

	RB0	Bac	Pse	MEAN(CMP)
CMP0	0.140 ab	0.138 ab	0.140 ab	0.139 B
CMP100	0.120 b	0.145 ab	0.145 ab	0.137 B
CMP200	0.136 ab	0.148 ab	0.160 a	0.148 A
CMP300	0.138 ab	0.138 ab	0.138 ab	0.138 B
MEAN(RB)	0.133 B	0.142 AB	0.145 A	

Lowercase letters indicate differences between means in interaction effects, whereas uppercase letters indicate differences between means in main effects. (P values: for average fruit weight; MEANcmp- 0.0234, MEANrb- < 0.0001, Int- < 0.0001). CMP0: 0 g m^− 2^ compost; CMP100: 100 g m^− 2^ compost; CMP200: 200 g m^− 2^ compost; CMP300: 300 g m^− 2^ compost; RB0: No PGPR; Bac: *Bacillus subtilis*; Pse: *Pseudomonas fluorescens*.

The effects of treatments on measured fruit quality parameters, excluding fruit firmness, fruit juice pH, fruit width, and length, were statistically insignificant. Fruit width varied between 3.28 and 3.59 cm, and fruit length varied between 15.16 and 16.95 cm (Supplementary Table 1). Regarding fruit colour, L value ranged between 37.95 and 35.30, a value between − 12.33 and − 13.97, and a b value between 20.76 and 17.87 (Supplementary Tables 2 and 3). The values of total soluble solids ranged from 3.17 to 3.83%, and titratable acidity ranged from 0.79 to 0.90 (Supplementary Table 4).

The average fruit firmness and pH of fruit juice were highest in the treatment without root bacteria, followed by the Bac and Pse treatments. When the interaction effect was analyzed, the highest flesh firmness was found in the CMP100 + RB0 interaction, with a value of 8.09 N. The lowest values were 6.64 and 6.53 N in the CMP200 + Pse and CMP300 + Pse treatments, respectively. As with juice pH, fruit firmness increased with increasing compost dose (Supplementary Table 5).

### Heatmap analysis

**Fig. 3:**
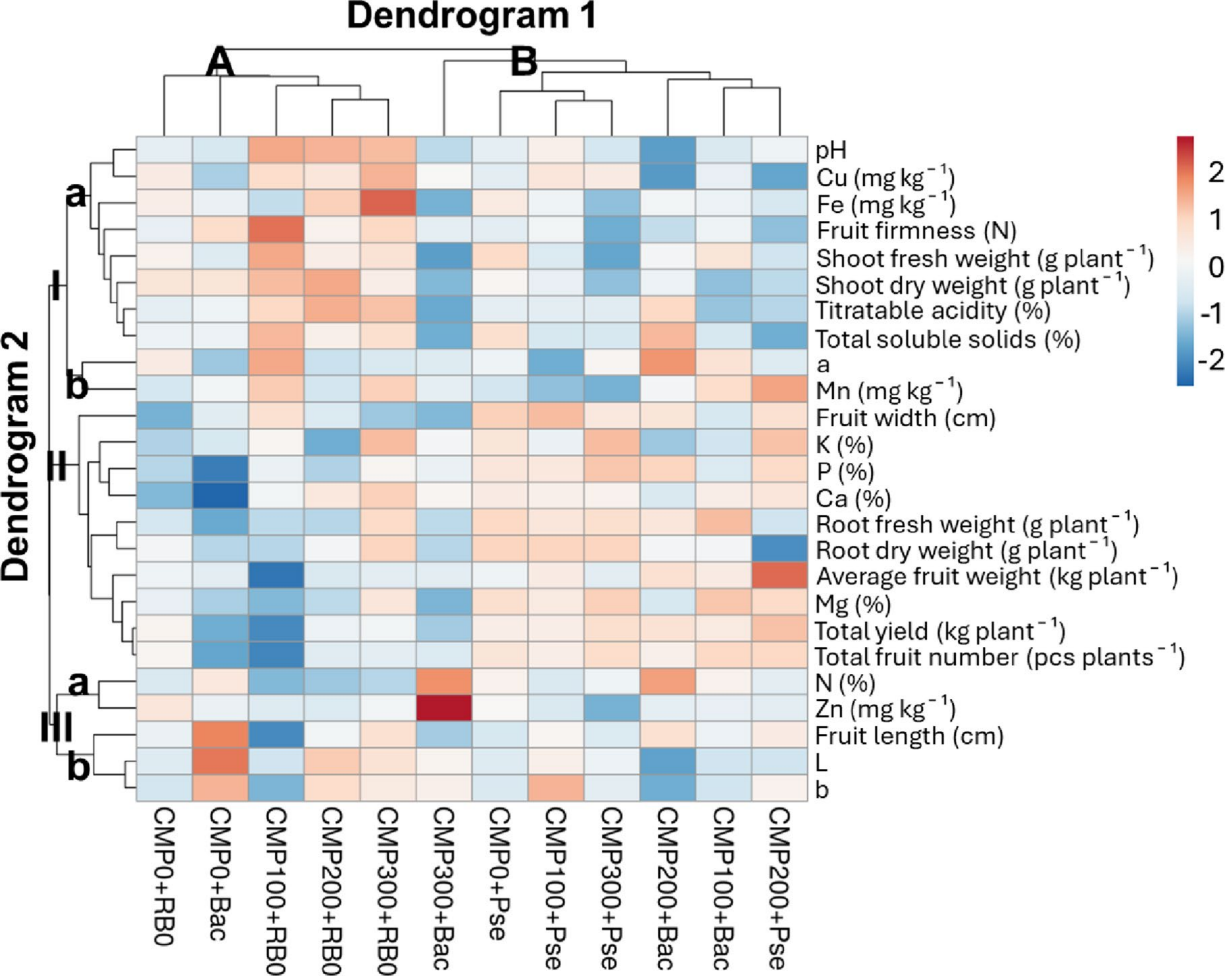
Heatmap analysis based on the given parameters.

An aggregate data clustering heatmap analysis was performed to facilitate a visual comparison of the effects of different compost and PGPR application rates on grafted cucumber cultivation under unheated greenhouse conditions. The results are presented in Fig. 3. The heatmap analysis yielded two dendrograms. Dendrogram 1 displays the experimental subjects, and Dendrogram 2 illustrates the parameters influencing the distribution. Two main groups were formed in the endrogram 1. The group on the left (A) included all compost doses without bacteria and CMP0 + Bac treatment, while the group on the right (B) included all bacteria treatments except one Bac treatment. When Dendogram 1 is analysed based on groups, group A stands out with high values in terms of L, b, dry and fresh weight of shoots, TA, pH, TSS, and fruit firmness, and low values in terms of yield parameters, root fresh and dry weight, fruit width, N, P, and Mg. While group B clustered with the opposite values of group A, CMP300 + Bac treatment differed significantly from the others. Group A, particularly the non-bacteria plots, was characterized by higher shoot biomass, pH, TSS, and firmness, but low yield. In contrast, Bacteria-treated plots, especially Pse (Group B), exhibited higher yields, root biomass, Mg, and P concentrations. In Dendrogram 2, the measured parameters were grouped to show the effects of the experimental subjects. The first group (I) of Dendrogram 2 was divided into two sub-clusters, while pH, Cu, Fe, fruit firmness, shoot fresh and dry weight, TA, and TSS were clustered in cluster I-a, and Mn was clustered in cluster I-b. In the second group, sub-cluster formation was lower than in the other groups, and fruit width, K, P, Ca, Mg, root fresh and dry weight, and yield parameters were clustered. The third group was again divided into two sub-clusters: N and Zn clustered in cluster III-a, and fruit length (L) and b clustered in cluster III-b. The clusters also confirm the effects on the subjects and on the measured parameters.

### Principal component analysis (PCA)

**Fig. 4:**
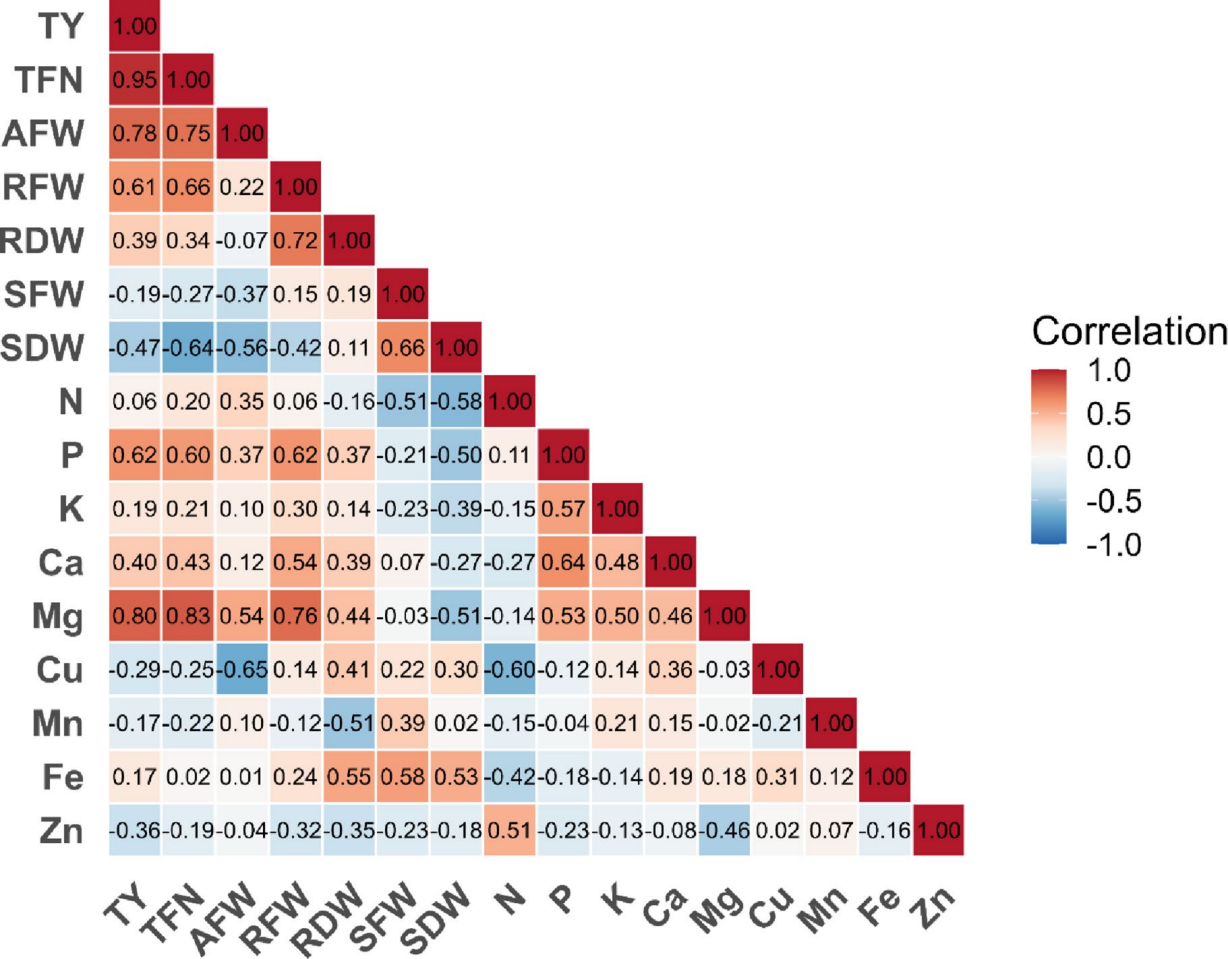
Correlation matrix showing the relationship between variables (TY: Total Yield, TFN: Total Fruit Number, AWF: Average Fruit Weight, RFW: Root Fresh Weight, RDW: Root Dry Weight, SFW: Shoot Fresh Weight, SDW: Shoot Dry Weight).

Principal Component Analysis (PCA) was applied to reveal relationships between plant growth and yield parameters and plant nutrient element contents, and to observe the effects of treatments. The correlation matrix, created to determine relationships among parameters, revealed patterns of diversity in the relationships between yield and plant growth parameters and nutrient elements (Fig. 4). In general, magnesium (Mg) plays a dominant role in yield, showing strong positive correlations with both total fruit number (*r* = 0.83) and average fruit weight (*r* = 0.80). Phosphorus (P) similarly exhibited positive relationships with total yield (*r* = 0.62), total fruit number (*r* = 0.6), and root dry weight (*r* = 0.62). Phosphorus and magnesium also showed positive correlations with the shoot biomass. On the other hand, the analysis also reveals noteworthy negative correlations. For example, a negative correlation was observed between copper (Cu) and average fruit weight, while a similar relationship was observed at the average level between copper (Cu) and nitrogen (N). This situation may indicate a possible antagonism between nutrient elements. Furthermore, a negative correlation was found between the shoot biomass and yield parameters.

The Principal Component Analysis (PCA) of the plant growth, yield, and element content parameters yielded five principal component (PC) axes with eigenvalues greater than 1 (Table 5). Factors with eigenvalues less than one were disregarded^67^. The five PC axes obtained had eigenvalues ranging from 5.59 to 1.19, accounting for 86.12% of the total variation. Upon detailed examination of the components, the first component (PC1, 34.97%) showed positive loadings with total yield, total fruit number, root fresh weight, Mg, and P contents. The second component (PC2, 22.64%) was associated with plant nutrient elements and shoot development, showing a positive relationship with shoot weights and Fe, and a negative relationship with N. The third component (PC3, 11.08%) was associated with average fruit weight, K, Ca, and Cu content. The fourth component (PC4, 10.52%) was particularly associated with Mn content and was negatively correlated with root dry weight. The fifth component (PC5, 6.91%) highlighted Zn content. These results support the findings that positive relationships exist between yield parameters and some nutrient elements, whereas some elements may have adverse effects, particularly on vegetative development.

**Table 5:** The PC axes resulting from PCA and the factor groups to which the variables belong.

	PC axis				
	1	2	3	4	5
Eigenvalues	5.59	3.62	1.77	1.68	1.11
Variation (%)	34.97	22.64	11.08	10.52	6.91
Total variation (%)	34.97	57.60	68.69	79.21	86.12
Parameters	Factor groups				
Total yield (kg plant^− 1^)	0.38	0.00	0.26	− 0.01	− 0.05
Total fruit number (pcs plant^− 1^)	0.39	− 0.06	0.16	− 0.06	0.05
Root fresh weight (g plant^− 1^)	0.33	0.19	− 0.02	− 0.11	0.21
P (%)	0.33	0.01	− 0.23	0.06	− 0.04
Mg (%)	0.37	0.12	0.07	0.10	− 0.12
Shoot fresh weight (g plant^− 1^)	− 0.11	0.37	0.24	0.25	0.20
Shoot dry weight (g plant^− 1^)	− 0.29	0.31	0.21	− 0.01	− 0.09
N (%)	0.06	− 0.41	0.05	− 0.21	0.35
Fe (mg/kg)	0.01	0.38	0.33	0.03	0.36
Average fruit weight (kg plant^− 1^)	0.28	− 0.23	0.34	0.17	0.05
K (%)	0.20	0.04	− 0.47	0.27	− 0.17
Ca (%)	0.24	0.21	− 0.32	0.17	0.26
Cu (mg kg^− 1^)	− 0.07	0.36	− 0.40	− 0.20	0.09
Root dry weight (g plant^− 1^)	0.19	0.34	0.02	− 0.40	0.12
Mn (mg kg)	− 0.06	0.00	0.00	0.73	0.21
Zn (mg kg)	− 0.13	− 0.25	− 0.20	− 0.06	0.68

**Fig. 5:**
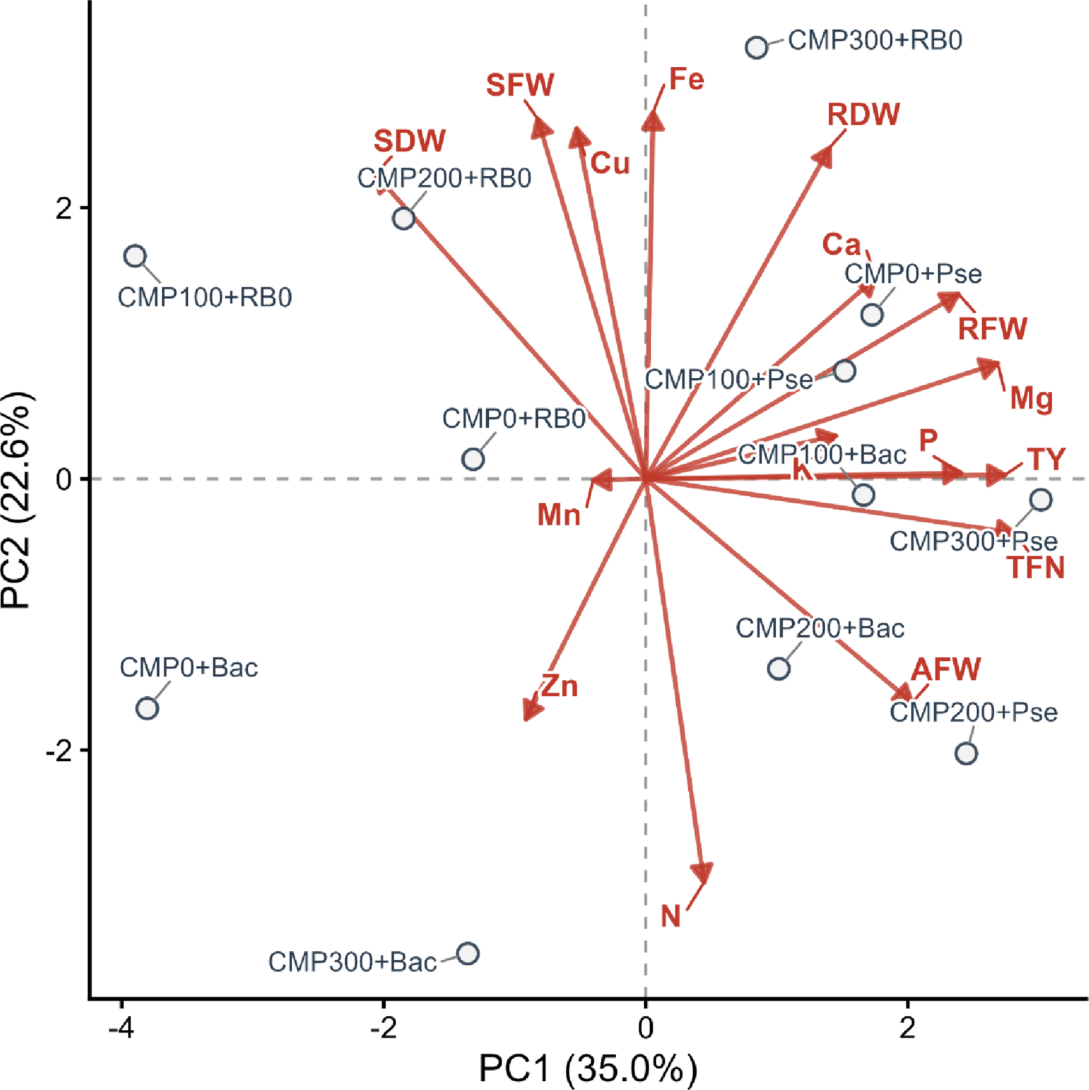
Biplot indicating the relationships between variables and applications created according to PC1 and PC2 (TY: Total Yield, TFN: Total Fruit Number, AWF: Average Fruit Weight, RFW: Root Fresh Weight, RDW: Root Dry Weight, SFW: Shoot Fresh Weight, SDW: Shoot Dry Weight).

A biplot was created to examine the relationships between the parameters and treatments in more detail, using the two components with the highest eigenvalues from the PCA results. These two components represented 57.6% of the total variation (Fig. 5). According to the PCA biplot, yield parameters (total yield, total number of fruits, and average fruit weight), some element contents (K, Ca, Mg), and root weights clustered on the positive axis of PC1, showing a positive correlation. This indicates that these parameters tend to increase together and are directly related to yield. On the negative axis, shoot weights were positioned, showing an opposite trend to the yield parameters. When examining PC2, biomass parameters and Cu and Fe were positively separated, while N, Zn, and average fruit weight were positioned on the negative axis.

When the applications were examined, it was determined that the Pse application exhibited high yield potential, particularly in yield parameters. In contrast, the Bac application showed a divergent trend in N and Zn content, parallel to the increase in compost application rate. In the control plots without bacterial application, a divergence in shoot weight was observed alongside compost applications. These results indicate that the applications exerted different effects on yield, plant growth parameters, and nutrient elements.

## Discussion

Today, with a new focus on ensuring agricultural sustainability, organic/biological fertilizers, especially compost, have gained significant importance, as they enhance soil fertility, improve soil structure, reduce erosion, suppress plant diseases, and minimize dependence on synthetic fertilizers^68^. However, the agronomic value of compost can be enhanced by enriching beneficial organisms^69^. This enrichment renders the compost biologically active, increasing agricultural and environmental benefits through the synergistic action of organic matter and biofertilizers^70^. Plant growth-promoting rhizobacteria solubilize and mobilize nutrients, converting them into forms that are readily accessible to plants. Moreover, they promote root and shoot development by producing phytohormones^71^. The presence of organic matter further supports the viability and persistence of these bacteria, both in the formulated product and within the rhizosphere^70^. Indeed, our study showed that compost, in combination with *P. fluorescens*, especially at 200–300 g m^−2^, improved yield by increasing fruit number and/or fruit weight, whereas compost alone primarily enhanced shoot biomass without further yield improvement. Magnesium was the key nutrient driving yield, while other macronutrients (e.g., phosphorus and calcium) played a minor role, and negative nutrient interactions may also influence yield dynamics.

In previous studies conducted on the compost use in cucumbers, shoot and root growth and biomass production increased with compost and vermicompost use^18^ while with compost treatment containing 75%, 100%, and 125% of the recommended N dose, with or without the addition of PGPR, fresh and dry shoot weights, as well as chlorophyll content, increased in cucumbers with 100% or 125% compost + PGPR application^43^. Amendments to different compost-origin treatments were also found to enhance plant growth under salt stress^72^ and water deficit^73^ conditions. The responses of beneficial organisms also vary according to strains and the availability of abiotic stressors^74^. For instance, *Bacillus* and *Pseudomonas* species can show antagonistic effects with other beneficial bacteria and fungi, but additional or synergistic effects may not always be observed^75^. In our study, the interaction of treatments affected root fresh and dry weights, whereas there were no significant differences in shoot growth. This outcome is consistent with greenhouse conditions in which fertigation, light, and temperature were maintained near optimal levels. Under such conditions, control plants likely approached a physiological ceiling for vegetative growth, meaning that biomass accumulation was constrained by non-nutritional factors rather than nutrient availability. As a result, the additional nutrients mobilized by PGPR were not invested in further shoot expansion but contributed instead to maintaining metabolic activity and supporting reproductive sinks, which are more responsive under high-input systems. This interpretation aligns with the observed improvement in marketable yield despite limited changes in shoot biomass. However, increased root growth was found to be related to the PGPRs, particularly in Pse, which shows extensive interactions with soil microorganisms that influence plant nutrition either directly—by enhancing nutrient availability and uptake—or indirectly, by promoting root growth^76^, supported by our results on nutrient uptake.

In our study, total yield per unit area ranged from 18 to 24.3 kg m^− 2^, and these values were within regional averages, changing between 10 and 40 kg m^− 2^^77^. Yield values are influenced by various factors, including the variety used, the use of grafted seedlings, climatic conditions, the length of growing period, number of plants per unit area, maintenance operations, and the emergence of diseases and/or pests^78^. In earlier studies, despite reports of increased cucumber yield with compost and PGPR, results varied according to the strain and plant species used^79^. For instance, Gül et al.^80^ showed that *the Pseudomonas putida* strain 18/1K and *Serratia marcescens* strain 62 yielded better than the control in naturally occurring Fusarium wilt (*Fusarium oxysporum* f.sp. *cucumerinum*). Ahamd et al.^81^ tested *P. fluorescens* with organic and mineral fertilisers. They reported that the treatments, alone or in combination with *P. fluorescens*, increased plant growth, yield, and fruit quality in cucumbers. The co-application of biogas fertiliser and *P. fluorescens* gave the best results. Zapata-Sifuentes et al.^82^ tested three bacterial species (*Pseudomonas paralactis* (KBendo6p7), *Sinorhizobium meliloti* (KBecto9p6), and *Acinetobacter radioresistens* (KBendo3p1)) in greenhouse cucumber cultivation. Inoculating with *Acinetobacter radioresistens* and *Sinorhizobium meliloti* resulted in better performance than the control. In our experiment, the yield was high, with increases in either fruit number or fruit weight observed in *P. fluorescens*, particularly with compost doses of 200 and 300 g m^−2^.This improvement was associated with increased uptake of significant elements such as magnesium (Mg) and phosphorus (P), as confirmed by our principal component analysis.

Compost and PGPR enhance plant growth through multiple mechanisms, including improved nutrient availability, modulation of phytohormone levels, and suppression of plant pathogens^45,83^. In our study, treatments had a powerful effect on plant nutrition. Leaf analysis showed that N content in cucumber leaves generally remained at adequate levels; however, N levels were higher in PGPR treatments. Bac treatments exhibited significantly higher nitrogen (N) levels than other treatments. Similar increases were obtained in melon^84^, sugarcane^85^, and cucumber with *B. velezensis* SX13^86^. It is observed that the P content exceeds the references in all treatments. Compared to the control treatments, P content was higher in compost doses (except CMP200), compost + PGPR interactions, and especially in the Pse treatment^81^. In wheat, N and P uptake increased with the application of *P. putida* and biochar^87^. It increased the amount of available P in melon^88^. Different results were obtained in N, P, and K contents when using the bacterial strain in cucumber^89^. It is understood that the K content in the leaves remained relatively low. However, the K value was sufficient in the area where the study was conducted, and fertilisation was made during cultivation. However, there was a general increase compared to the control treatments, and higher values were observed in the Pse treatment. Ca and Mg contents in cucumber leaf tissue were above the reference values and increased with the treatments. Copper, iron, and zinc concentrations in the leaves were also within the reference values; manganese concentration was slightly below the sufficient value.

The study further evaluated the relationships between plant growth, yield, and nutrient element contents using a correlation matrix and Principal Component Analysis (PCA) to identify nutrients most associated with yield. The results revealed the critical roles of nutrient elements and the different effects of treatments. In the correlation matrix, positive correlations between Mg and yield parameters indicate that Mg affects yield. This can be explained by Mg’s central role in plant physiology^90^. Therefore, adequate Mg levels may have increased both fruit number and weight by supporting the production and transport of carbohydrates necessary for fruit development and filling^91^. Similarly, P showed a positive correlation with total yield, fruit number, and root development. P is vital for energy metabolism (ATP), nucleic acid synthesis, and root development^92,93^. A robust root system has positively influenced final yield by increasing the plant’s capacity to take up water and nutrients.

On the other hand, negative correlations between Cu and average fruit weight and nitrogen (N) suggest that Cu may exhibit antagonistic effects at specific concentrations, possibly due to beneficial bacteria and compost applications. The literature emphasises that copper, particularly at high levels, can negatively affect protein synthesis and nitrogen metabolism^94^. Similarly, the negative relationship observed between shoot biomass and yield parameters indicates competition for natural resources between vegetative and generative development. This relationship reflects the imbalance between vegetative and generative growth frequently observed in plants^95,96^. This result highlights that intensive shoot growth may not always positively impact yield. The negative correlation between N and plant weight appears to be related to N dilution and a decreased N ratio, resulting from increased biomass from compost application without bacteria. Indeed, previous studies have mentioned this effect^97,98^. Additionally, increased plant nutrient concentrations in conjunction with compost and bacterial applications may not directly reflect plant growth parameters^99,100^. It should be noted that the correlation results provided are only an indication of linear relationships between variables and do not provide any inference regarding causality. Therefore, when interpreting the findings, the limitations of correlation analysis were taken into account, and the results were used only as a guiding indicator without any claim of causality.

The biplot graph has more clearly revealed the position of the treatments within these relationships. The clustering of Pse applications by yield parameters, demonstrating high yield potential, is consistent with the known growth-promoting effects of this bacterium^88,101,102^. The literature reports that Pse increases yield by promoting phytohormone production, siderophore synthesis, and root development^103^. In contrast, the differentiation of Bac applications in terms of N and Zn contents, parallel to compost doses, reflects the effects of this species, particularly on the mobilisation and uptake of nutrient elements^100,104,105^. Previous studies have also confirmed that Bac increases Zn solubility and contributes to nitrogen uptake through organic acid and siderophore production^106,107^. In this context, the findings suggest that Bac may be more effective at enriching nutrient content than at directly increasing yield.

The fact that compost applications without bacterial inoculation promote shoot biomass indicates that organic matter addition particularly stimulates vegetative growth in this study. However, when compared to bacterial applications, this did not directly translate into yield; instead, it was negatively correlated with yield parameters in PCA. This finding indicates that organic matter alone may not be sufficient to enhance generative performance, and that a more balanced effect can be achieved when combined with biological inoculants^70,108–110^.

A range of interrelated factors, such as genetic material, climatic conditions, production techniques, agronomic practices, cultural management, stress, harvesting time, and post-harvest operations, influences fruit quality^111,112^. In this experiment, fruit quality parameters, excluding fruit firmness and fruit juice pH, did not change significantly. The findings on fruit quality are inconsistent. Abou-El-Hassan et al.^43^ reported that total soluble solids reached their highest value at the highest compost dose (125%) and with PGPR application. When *Pseudomonas paralactis*, *Sinorhizobium meliloti*, and *Acinetobacter radioresistens* were tested as root bacteria in cucumber cultivation, it was determined that *S. meliloti* increased the content of phenolic compounds, flavonoids, and antioxidant capacity^82^. Among the 17 Streptomyces strains tested, SS12 reduced nitrate and reducing sugars, while increasing antioxidant content and firmness^89^. *P. putida* P3-57 increased the overall acceptance score of fruit based on higher aroma, flavour, and juice score^113^. However, there was no significant effect of root bacteria on fruit size, colour, TSS, and acidity values of cucumber on the fruit quality characteristics examined in our study. Similar to previous studies on melon^84^ and cucumber^79^, no significant effect of root bacteria on fruit quality was found. We think that the changes in fruit firmness and pH could be related to soil moisture^114^.

In future studies, more frequent monitoring of plant growth and quality analyses are expected to reveal differences across plant growth stages. This single-cycle study was conducted under a regular fertigation regime, which may have moderated the full expression of biostimulant effects and thus warrants validation under lower-input scenarios. Despite these constraints, the results consistently highlight the synergistic potential of compost and microbial inoculants under commercial greenhouse conditions.

## Conclusion

The findings of this study confirm the substantial benefits of incorporating biostimulants into standard high-input production systems. Compost application—notably when combined with *Pseudomonas fluorescens*—significantly increased marketable yield, demonstrating its effectiveness in improving soil structure and supporting key nutrient pools. The presence of PGPRs amplified these gains by enhancing nutrient uptake, indicating a clear additive interaction between compost inputs and microbial inoculants. Together, these outcomes provide meaningful evidence to improve existing fertilization strategies in controlled-environment agriculture. Future research should focus on optimizing dose and application timing and evaluating these strategies in systems with reduced synthetic inputs, with the long-term objective of developing practical, sustainable recommendations for protected cropping operations. Examination under stress conditions, such as salt or water stress, would also be beneficial.

## Supplementary Information

Below is the link to the electronic supplementary material.

Supplementary Material 1

## Data Availability

Data will be made available from the corresponding author upon request.

## Declarations

### Permissions and compliance statement

The experiments were conducted by the authors, and no external permissions were required for data collection. All biological materials and commercial products used in this study were properly licensed and complied with applicable regulations.
