# Supplementary material for: Enhancing cucumber production through compost and plant growth promoting rhizobacteria in an unheated soil based greenhouse
Source: Sci Rep. 2026 Jan 30;16:6742. doi: 10.1038/s41598-026-36907-2 (PMC12914054; doi:10.1038/s41598-026-36907-2)
Supplement: Supplementary file 1 — Supplementary Material 1 [file 41598_2026_36907_MOESM1_ESM.docx]

**Enhancing Cucumber Production through Compost and Plant Growth Promoting Rhizobacteria in an Unheated Soil Based Greenhouse** – Supplementary Material

**Table 1.**

Effects of treatments on fruit width (cm) and fruit length (cm)

|  | Fruit width (cm) | | | | Fruit length (cm) | | | |
| --- | --- | --- | --- | --- | --- | --- | --- | --- |
|  | RB0 | Bac | Pse | MEAN(CMP) | RB0 | Bac | Pse | MEAN(CMP) |
| CMP0 | 3.28 | 3.39 | 3.53 | 3.40 | 16.01 | 16.95 | 15.82 | 16.26 |
| CMP100 | 3.50 | 3.37 | 3.55 | 3.47 | 15.16 | 16.05 | 16.18 | 15.8 |
| CMP200 | 3.38 | 3.59 | 3.50 | 3.46 | 16.1 | 16.44 | 16.31 | 16.28 |
| CMP300 | 3.30 | 3.29 | 3.48 | 3.36 | 16.45 | 15.58 | 15.88 | 15.97 |
| MEAN(RB) | 3.37 | 3.39 | 3.51 |  | 15.93 | 16.26 | 16.04 |  |

P values: for fruit width; MEANcmp- 0.1161, MEANrb- 0.0047, Int- 0.3368; for fruit length; MEANcmp- 0.4307, MEANrb- 0.5385, Int- 0.2091. CMP0: 0 g m^-2^ compost; CMP100: 100 g m^-2^ compost; CMP200: 200 g m^-2^ compost; CMP300: 300 g m^-2^ compost; RB0: No PGPR; Bac: *Bacillus subtilis*; Pse: *Pseudomonas fluorescens*.

**Table 2.**

Effect of treatments on L value

|  | RB0 | Bac | Pse | MEAN(CMP) |
| --- | --- | --- | --- | --- |
| CMP0 | 36.21 | 37.95 | 36.22 | 36.79 |
| CMP100 | 35.97 | 35.98 | 36.78 | 36.24 |
| CMP200 | 37.33 | 35.30 | 35.98 | 36.20 |
| CMP300 | 37.02 | 36.71 | 36.45 | 36.72 |
| MEAN(RB) | 36.63 | 36.48 | 36.35 |  |

P values: for lightness; MEANcmp- 0.5504, MEANrb- 0.8267, Int- 0.1152. CMP0: 0 g m^-2^ compost; CMP100: 100 g m^-2^ compost; CMP200: 200 g m^-2^ compost; CMP300: 300 g m^-2^ compost; RB0: No PGPR; Bac: *Bacillus subtilis*; Pse: *Pseudomonas fluorescens*.

**Table 3.**

Effect of applications on green-red (a) and blue-yellow (b) opponent color.

|  | a | | | | b | | | |
| --- | --- | --- | --- | --- | --- | --- | --- | --- |
|  | RB0 | Bac | Pse | MEAN(CMP) | RB0 | Bac | Pse | MEAN(CMP) |
| CMP0 | -12.96 | -13.80 | -13.24 | -13.33 | 18.69 | 20.76 | 18.83 | 19.43 |
| CMP100 | -12.45 | -12.84 | -13.97 | -13.08 | 18.00 | 18.65 | 20.75 | 19.13 |
| CMP200 | -13.62 | -12.33 | -13.42 | -13.12 | 20.24 | 17.87 | 19.66 | 19.26 |
| CMP300 | -13.48 | -13.41 | -13.11 | -13.33 | 19.86 | 19.74 | 19.04 | 19.54 |
| MEAN(RB) | -13.12 | -13.09 | -13.43 |  | 19.20 | 19.26 | 19.57 |  |

P values: for a; MEANcmp- 0.7963, MEANrb- 0.4109. Int- 0.0404; for b; MEANcmp- 0.9318, MEANrb- 0.7947, Int- 0.0472. CMP0: 0 g m^-2^ compost; CMP100: 100 g m^-2^ compost; CMP200: 200 g m^-2^ compost; CMP300: 300 g m^-2^ compost; RB0: No PGPR; Bac: *Bacillus subtilis*; Pse: *Pseudomonas fluorescens*.

**Table 4.**

The effect of treatments on total soluble solids and titratable acidity.

|  | Total Soluble Solids (%) | | | | Titratable Acidity (mval 100ml^-1^) | | | |
| --- | --- | --- | --- | --- | --- | --- | --- | --- |
|  | RB0 | Bac | Pse | MEAN(CMP) | RB0 | Bac | Pse | MEAN(CMP) |
| CMP0 | 3.53 | 3.48 | 3.71 | 3.57 | 0.84 | 0.85 | 0.84 | 0.84 |
| CMP100 | 3.77 | 3.39 | 3.36 | 3.51 | 0.89 | 0.80 | 0.84 | 0.84 |
| CMP200 | 3.60 | 3.83 | 3.17 | 3.53 | 0.91 | 0.89 | 0.81 | 0.87 |
| CMP300 | 3.68 | 3.21 | 3.44 | 3.45 | 0.90 | 0.79 | 0.84 | 0.84 |
| MEAN(RB) | 3.65 | 3.48 | 3.42 |  | 0.89 | 0.83 | 0.83 |  |

P values: For TSS; MEANcmp- 0.9684, MEANrb- 0.5979, Int- 0.7503; for TA; MEANcmp- 0.7619, MEANrb- 0.0991, Int- 0.5200. CMP0: 0 g m^-2^ compost; CMP100: 100 g m^-2^ compost; CMP200: 200 g m^-2^ compost; CMP300: 300 g m^-2^ compost; RB0: No PGPR; Bac: *Bacillus subtilis*; Pse: *Pseudomonas fluorescens*.

**Table 5.**

Effect of treatments on fruit firmness and pH of fruit juice

|  | Fruit firmness (N) | | | | pH | | | |
| --- | --- | --- | --- | --- | --- | --- | --- | --- |
|  | RB0 | Bac | Pse | MEAN(CMP) | RB0 | Bac | Pse | MEAN(CMP) |
| CMP0 | 7.10 ab | 7.58 ab | 7.06 ab | 7.25 A | 5.71 ac | 5.70 abc | 5.71 abc | 5.70 A |
| CMP100 | 8.09 a | 7.18 ab | 7.19 ab | 7.48 A | 5.78 a | 5.70 abc | 5.73 abc | 5.74 A |
| CMP200 | 7.34 ab | 6.82 ab | 6.64 b | 6.94 A | 5.77 ab | 5.65 c | 5.72 abc | 5.71 A |
| CMP300 | 7.62 ab | 7.07 ab | 6.53 b | 7.08 A | 5.77 ab | 5.68 bc | 5.69 abc | 5.72 A |
| MEAN(RB) | 7.54 A | 7.16 AB | 6.86 B |  | 5.76 A | 5.68B | 5.71 B |  |

Lowercase letters indicate differences between means in interaction effects, whereas uppercase letters indicate differences between means in main effects. (P values: for firmness; MEANcmp- 0.016, MEANrb- 0.0034, Int- 0.1970; for pH; MEANcmp- 0.2056, MEANrb- < 0.0001, Int- 0.1216). CMP0: 0 g m^-2^ compost; CMP100: 100 g m^-2^ compost; CMP200: 200 g m^-2^ compost; CMP300: 300 g m^-2^ compost; RB0: No PGPR; Bac: *Bacillus subtilis*; Pse: *Pseudomonas fluorescens*.
